# 1-(2,4-Dichloro­phen­yl)-5-(2-nitro­anilino)-1*H*-pyrazole-4-carbonitrile

**DOI:** 10.1107/S1600536812011506

**Published:** 2012-03-24

**Authors:** Ju Liu, Zhi-Qiang Cai, Yang Wang, Chun-Yan Li, Li-Feng Xu

**Affiliations:** a College of Pharmacy, Liaoning University, Shenyang 110036, People’s Republic of China; b Tianjin Key Laboratory of Molecular Design and Drug Discovery, State Key Laboratory of Drug Delivery Technology and Pharmacokinetics, Tianjin Institute of Pharmaceutical Research, Tianjin 300193, People’s Republic of China; c Shenlong Pharmaceutical Limited Company, Shenyang 110141, People’s Republic of China

## Abstract

In the title compound, C_16_H_9_Cl_2_N_5_O_2_, the folded mol­ecular conformation is characterized by a dihedral angle between the two benzene rings of 74.03 (5)°. An intra­molecular N—H⋯O hydrogen bond is observed between the H atom of the amide group and a nitro-group O atom. Inter­molecular C—H⋯O and N—H⋯N hydrogen bonds feature in the crystal packing.

## Related literature

For general background to *N-*1-diaryl-1*H*-pyrazol-5-amine derivatives as synthetic inter­mediates in the preparation of medicinal compounds and the synthesis of the title compound, see: Markwalder *et al.* (2004 ▶); Mehdi *et al.* (2010 ▶). For the pharmacological activity of the 5-amino­pyrazole nucleus, see: Nils *et al.* (2010 ▶); Aymn *et al.* (2005 ▶).
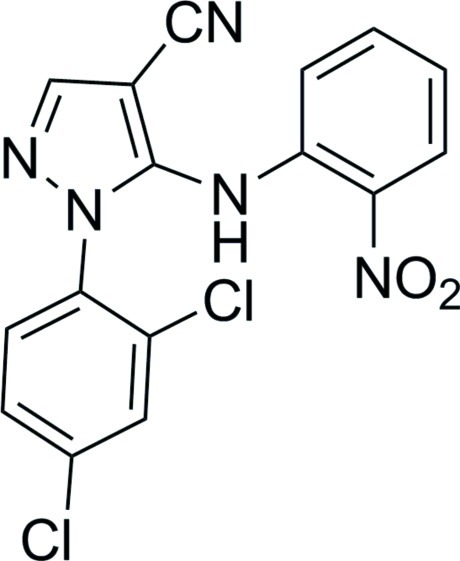


## Experimental

### 

#### Crystal data

C_16_H_9_Cl_2_N_5_O_2_
*M* *_r_* = 374.18
Orthorhombic, 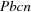

*a* = 13.878 (3) Å
*b* = 13.475 (3) Å
*c* = 17.421 (4) Å
*V* = 3257.7 (11) Å^3^
*Z* = 8
Mo *K*α radiation
μ = 0.42 mm^−1^
*T* = 293 K
0.20 × 0.18 × 0.12 mm

#### Data collection

Rigaku Saturn724 CCD diffractometer
Absorption correction: multi-scan (*CrystalClear*; Rigaku/MSC, 2005) ▶ *T*_min_ = 0.921, *T*_max_ = 0.951
31011 measured reflections
3888 independent reflections
2738 reflections with *I* > 2σ(*I*)
*R*_int_ = 0.050

#### Refinement

*R*[*F* ^2^ > 2σ(*F* ^2^)] = 0.052
*wR*(*F* ^2^) = 0.142
*S* = 1.04
3888 reflections
231 parameters
H atoms treated by a mixture of independent and constrained refinement
Δρ_max_ = 0.24 e Å^−3^
Δρ_min_ = −0.24 e Å^−3^

### 

Data collection: *RAPID-AUTO* (Rigaku, 1998 ▶); cell refinement: *RAPID-AUTO*; data reduction: *CrystalClear* (Rigaku/MSC, 2005) ▶; program(s) used to solve structure: *SHELXS97* (Sheldrick, 2008 ▶); program(s) used to refine structure: *SHELXL97* (Sheldrick, 2008 ▶); molecular graphics: *SHELXTL* (Sheldrick, 2008 ▶); software used to prepare material for publication: *SHELXTL*.

## Supplementary Material

Crystal structure: contains datablock(s) I, global. DOI: 10.1107/S1600536812011506/kp2396sup1.cif

Structure factors: contains datablock(s) I. DOI: 10.1107/S1600536812011506/kp2396Isup2.hkl

Supplementary material file. DOI: 10.1107/S1600536812011506/kp2396Isup3.cml

Additional supplementary materials:  crystallographic information; 3D view; checkCIF report

## Figures and Tables

**Table 1:** Hydrogen-bond geometry (Å, °)

*D*—H⋯*A*	*D*—H	H⋯*A*	*D*⋯*A*	*D*—H⋯*A*
C9—H9⋯O2^i^	0.93	2.51	3.050 (3)	117
C5—H5⋯O1^ii^	0.93	2.58	3.374 (3)	143
N4—H4⋯N3^iii^	0.82 (3)	2.62 (3)	3.201 (3)	129 (2)
N4—H4⋯O1	0.82 (3)	2.02 (3)	2.608 (2)	128 (2)

Symmetry codes: (i) 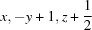; (ii) 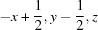; (iii) 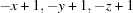.

**Fig. 1.**
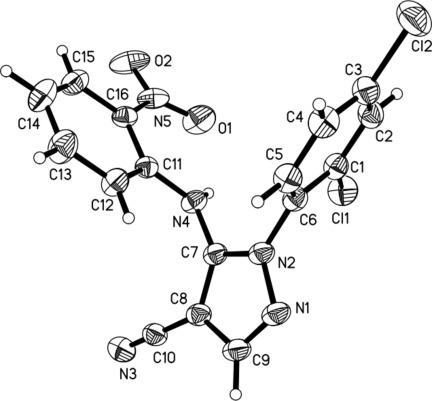
The structure of C16H9Cl2N5O2 with all non-H atom-labelling scheme and ellipsoids drawn at the 50% probability level.

## supplementary crystallographic information

### Comment

*N*-1-Diaryl-1*H*-pyrazol-5-amine derivatives possess therapeutic value. They are synthetic intermediates in the preparation of medicinal compounds (Markwalder *et al.*, 2004; Mehdi *et al.*, 2010). 5-Aminopyrazole nucleus is associated with several pharmacological activities such as selective adenosine A1 receptor antagonists (Nils *et al.*, 2010) and antimicrobial activity (Aymn *et al.*, 2005). In view of this biological importance a part of our ongoing studies of pyrazole derivatives includes the crystal structure determination of the title compound. The folded molecular conformation is characterised by the dihedral angle between the two benzene rings of 74.03 (5) ° (Fig. 1). Intramolecular N—H···O hydrogen bond between the hydrogen of the amide group and the nitro group O atom is observed. The crystal packing is stabilised by intermolecular C—H···O and N—H···N hydrogen bonds (Table 1).

### Experimental

To a mixture of 5-amino-1-(2,4-dichlorophenyl)-1*H*-pyrazole-4-carbonitrile (3.0 g, 11.85 mmol) and *o*-nitrochlorobenzene (3.74 g, 23.74 mmol) in dimethyl sulfoxide (10 mL) lithium hydroxide monohydrate (0.74 g, 17.87 mmol) was added. The solution was heated at 343 K for 4.5 h. After cooling to 293 K, 50 mL of water was added to the reaction mixture. The resulting precipitate was collected by filtration, washed with ethanol to yield the title compound as a brown yellow solid (3.28 g, 73.95%). Crystals suitable for X-ray analysis were obtained from ethanol:acetone (1:1) solution by slow evaporation.

### Refinement

All H atoms were geometrically positioned (C—H 0.93–0.98 Å) and treated as riding, with *U*_iso_(H) = 1.2Ueq(C).

### Crystal data

**Table:**

C_16_H_9_Cl_2_N_5_O_2_	*F*(000) = 1520
*M**_r_* = 374.18	*D*_x_ = 1.526 Mg m^−^^3^
Orthorhombic, *P**b**c**n*	Mo *K*α radiation, λ = 0.71073 Å
Hall symbol: -P2n2ab	Cell parameters from 7017 reflections
*a* = 13.878 (3) Å	θ = 2.4–28.0°
*b* = 13.475 (3) Å	µ = 0.42 mm^−^^1^
*c* = 17.421 (4) Å	*T* = 293 K
*V* = 3257.7 (11) Å^3^	Prism, yellow
*Z* = 8	0.20 × 0.18 × 0.12 mm

### Data collection

**Table:**

Rigaku Saturn724 CCD diffractometer	3888 independent reflections
Radiation source: rotating anode	2738 reflections with *I* > 2σ(*I*)
Multilayer monochromator	*R*_int_ = 0.050
Detector resolution: 7.31 pixels mm^-1^	θ_max_ = 27.9°, θ_min_ = 2.3°
ω scans	*h* = −17→17
Absorption correction: multi-scan (*CrystalClear*; Rigaku/MSC, 2005)	*k* = −17→16
*T*_min_ = 0.921, *T*_max_ = 0.951	*l* = −22→22
31011 measured reflections	

### Refinement

**Table:**

Refinement on *F*^2^	Secondary atom site location: difference Fourier map
Least-squares matrix: full	Hydrogen site location: inferred from neighbouring sites
*R*[*F*^2^ > 2σ(*F*^2^)] = 0.052	H atoms treated by a mixture of independent and constrained refinement
*wR*(*F*^2^) = 0.142	*w* = 1/[σ^2^(*F*_o_^2^) + (0.0759*P*)^2^] where *P* = (*F*_o_^2^ + 2*F*_c_^2^)/3
*S* = 1.04	(Δ/σ)_max_ = 0.001
3888 reflections	Δρ_max_ = 0.24 e Å^−^^3^
231 parameters	Δρ_min_ = −0.24 e Å^−^^3^
0 restraints	Extinction correction: *SHELXL97* (Sheldrick, 2008), Fc^*^=kFc[1+0.001xFc^2^λ^3^/sin(2θ)]^-1/4^
Primary atom site location: structure-invariant direct methods	Extinction coefficient: 0.0166 (13)

### Special details

**Table:**

Geometry. All e.s.d.'s (except the e.s.d. in the dihedral angle between two l.s. planes) are estimated using the full covariance matrix. The cell e.s.d.'s are taken into account individually in the estimation of e.s.d.'s in distances, angles and torsion angles; correlations between e.s.d.'s in cell parameters are only used when they are defined by crystal symmetry. An approximate (isotropic) treatment of cell e.s.d.'s is used for estimating e.s.d.'s involving l.s. planes.
Refinement. Refinement of *F*^2^ against ALL reflections. The weighted *R*-factor *wR* and goodness of fit *S* are based on *F*^2^, conventional *R*-factors *R* are based on *F*, with *F* set to zero for negative *F*^2^. The threshold expression of *F*^2^ > σ(*F*^2^) is used only for calculating *R*-factors(gt) *etc*. and is not relevant to the choice of reflections for refinement. *R*-factors based on *F*^2^ are statistically about twice as large as those based on *F*, and *R*- factors based on ALL data will be even larger.

### Fractional atomic coordinates and isotropic or equivalent isotropic displacement parameters (Å^2^)

**Table:**

	*x*	*y*	*z*	*U*_iso_*/*U*_eq_	
Cl1	0.15552 (5)	0.60779 (4)	0.46994 (4)	0.0673 (2)	
Cl2	−0.14562 (5)	0.48959 (6)	0.30041 (5)	0.0881 (3)	
O1	0.35014 (15)	0.63038 (12)	0.30863 (12)	0.0858 (6)	
O2	0.41521 (15)	0.62525 (14)	0.19700 (10)	0.0914 (6)	
N1	0.20172 (14)	0.33598 (13)	0.53423 (10)	0.0540 (5)	
N2	0.20985 (13)	0.39203 (11)	0.46920 (9)	0.0467 (4)	
N3	0.54146 (17)	0.37889 (14)	0.51865 (12)	0.0667 (5)	
N4	0.32727 (13)	0.46781 (13)	0.38776 (9)	0.0477 (4)	
N5	0.37959 (14)	0.58365 (14)	0.25298 (11)	0.0609 (5)	
C1	0.09266 (15)	0.51444 (13)	0.42412 (12)	0.0481 (5)	
C2	0.00946 (15)	0.53705 (15)	0.38503 (13)	0.0558 (6)	
H2A	−0.0128	0.6021	0.3830	0.067*	
C3	−0.04013 (16)	0.46247 (16)	0.34907 (13)	0.0556 (6)	
C4	−0.00852 (16)	0.36558 (16)	0.35107 (13)	0.0575 (6)	
H4A	−0.0428	0.3158	0.3261	0.069*	
C5	0.07443 (15)	0.34366 (14)	0.39045 (12)	0.0521 (5)	
H5	0.0962	0.2785	0.3925	0.063*	
C6	0.12581 (15)	0.41746 (14)	0.42708 (11)	0.0446 (5)	
C7	0.30239 (15)	0.41365 (13)	0.45245 (11)	0.0438 (5)	
C8	0.35782 (15)	0.37153 (15)	0.50928 (12)	0.0476 (5)	
C9	0.29092 (16)	0.32434 (15)	0.55794 (11)	0.0536 (5)	
H9	0.3079	0.2890	0.6018	0.064*	
C10	0.45966 (19)	0.37500 (15)	0.51458 (12)	0.0516 (5)	
C11	0.34423 (13)	0.42402 (14)	0.31790 (11)	0.0407 (4)	
C12	0.33508 (16)	0.32211 (15)	0.30894 (12)	0.0559 (6)	
H12	0.3164	0.2838	0.3508	0.067*	
C13	0.35285 (19)	0.27656 (18)	0.24013 (15)	0.0735 (7)	
H13	0.3455	0.2082	0.2360	0.088*	
C14	0.3815 (2)	0.3305 (2)	0.17670 (14)	0.0750 (7)	
H14	0.3942	0.2988	0.1304	0.090*	
C15	0.39084 (16)	0.4299 (2)	0.18286 (12)	0.0608 (6)	
H15	0.4098	0.4670	0.1404	0.073*	
C16	0.37242 (14)	0.47708 (15)	0.25204 (11)	0.0453 (5)	
H4	0.3360 (18)	0.528 (2)	0.3926 (15)	0.077 (8)*	

### Atomic displacement parameters (Å^2^)

**Table:**

	*U*^11^	*U*^22^	*U*^33^	*U*^12^	*U*^13^	*U*^23^
Cl1	0.0852 (5)	0.0448 (3)	0.0719 (4)	−0.0062 (3)	−0.0050 (3)	−0.0152 (3)
Cl2	0.0593 (4)	0.1038 (6)	0.1013 (6)	0.0108 (3)	−0.0192 (4)	−0.0065 (4)
O1	0.1372 (18)	0.0455 (10)	0.0746 (13)	−0.0036 (9)	0.0066 (11)	0.0082 (9)
O2	0.1296 (16)	0.0868 (13)	0.0577 (11)	−0.0414 (11)	−0.0163 (11)	0.0338 (9)
N1	0.0691 (13)	0.0515 (10)	0.0413 (9)	−0.0029 (8)	0.0045 (8)	0.0103 (7)
N2	0.0577 (11)	0.0429 (9)	0.0394 (9)	−0.0014 (7)	0.0013 (7)	0.0059 (6)
N3	0.0697 (15)	0.0613 (12)	0.0691 (14)	0.0055 (10)	−0.0084 (10)	0.0057 (9)
N4	0.0668 (11)	0.0363 (9)	0.0401 (9)	−0.0063 (8)	0.0028 (8)	0.0020 (7)
N5	0.0745 (13)	0.0594 (12)	0.0488 (12)	−0.0166 (10)	−0.0150 (10)	0.0172 (9)
C1	0.0547 (13)	0.0399 (10)	0.0499 (12)	−0.0017 (9)	0.0047 (10)	−0.0043 (8)
C2	0.0593 (14)	0.0464 (12)	0.0617 (14)	0.0075 (10)	0.0046 (11)	−0.0014 (10)
C3	0.0485 (13)	0.0607 (14)	0.0576 (14)	0.0001 (10)	0.0000 (10)	0.0011 (10)
C4	0.0570 (14)	0.0552 (13)	0.0603 (14)	−0.0137 (11)	0.0003 (11)	−0.0063 (10)
C5	0.0603 (13)	0.0374 (10)	0.0586 (13)	−0.0049 (9)	0.0041 (11)	−0.0001 (9)
C6	0.0504 (12)	0.0412 (10)	0.0421 (11)	−0.0031 (8)	0.0036 (9)	0.0022 (8)
C7	0.0556 (13)	0.0383 (10)	0.0375 (10)	0.0000 (8)	0.0012 (9)	0.0007 (7)
C8	0.0616 (14)	0.0411 (10)	0.0400 (11)	0.0023 (9)	−0.0034 (9)	−0.0011 (8)
C9	0.0733 (16)	0.0472 (12)	0.0401 (11)	0.0021 (10)	−0.0014 (10)	0.0068 (8)
C10	0.0625 (16)	0.0450 (11)	0.0472 (12)	0.0066 (10)	−0.0054 (10)	0.0006 (8)
C11	0.0394 (10)	0.0420 (10)	0.0406 (10)	−0.0025 (8)	−0.0020 (8)	0.0023 (8)
C12	0.0729 (14)	0.0439 (11)	0.0511 (13)	−0.0054 (10)	0.0077 (11)	−0.0017 (9)
C13	0.097 (2)	0.0552 (15)	0.0684 (17)	−0.0038 (12)	0.0093 (14)	−0.0156 (12)
C14	0.0862 (18)	0.0849 (19)	0.0540 (15)	−0.0084 (15)	0.0118 (13)	−0.0203 (13)
C15	0.0567 (14)	0.0836 (17)	0.0422 (12)	−0.0094 (12)	0.0042 (10)	0.0003 (11)
C16	0.0428 (11)	0.0514 (11)	0.0419 (11)	−0.0071 (9)	−0.0053 (8)	0.0061 (8)

### Geometric parameters (Å, º)

**Table:**

Cl1—C1	1.726 (2)	C4—C5	1.372 (3)
Cl2—C3	1.731 (2)	C4—H4A	0.9300
O1—N5	1.226 (3)	C5—C6	1.380 (3)
O2—N5	1.229 (2)	C5—H5	0.9300
N1—C9	1.314 (3)	C7—C8	1.376 (3)
N1—N2	1.366 (2)	C8—C9	1.409 (3)
N2—C7	1.349 (3)	C8—C10	1.417 (3)
N2—C6	1.420 (3)	C9—H9	0.9300
N3—C10	1.139 (3)	C11—C12	1.388 (3)
N4—C11	1.373 (2)	C11—C16	1.407 (3)
N4—C7	1.386 (2)	C12—C13	1.369 (3)
N4—H4	0.82 (3)	C12—H12	0.9300
N5—C16	1.440 (3)	C13—C14	1.381 (3)
C1—C2	1.375 (3)	C13—H13	0.9300
C1—C6	1.386 (3)	C14—C15	1.350 (3)
C2—C3	1.370 (3)	C14—H14	0.9300
C2—H2A	0.9300	C15—C16	1.386 (3)
C3—C4	1.378 (3)	C15—H15	0.9300
			
C9—N1—N2	104.40 (16)	N2—C7—C8	106.71 (18)
C7—N2—N1	112.17 (16)	N2—C7—N4	121.77 (18)
C7—N2—C6	128.19 (16)	C8—C7—N4	131.51 (19)
N1—N2—C6	119.60 (17)	C7—C8—C9	104.52 (19)
C11—N4—C7	122.49 (17)	C7—C8—C10	126.21 (19)
C11—N4—H4	119.2 (18)	C9—C8—C10	129.26 (19)
C7—N4—H4	118.1 (18)	N1—C9—C8	112.19 (18)
O1—N5—O2	121.8 (2)	N1—C9—H9	123.9
O1—N5—C16	119.88 (18)	C8—C9—H9	123.9
O2—N5—C16	118.3 (2)	N3—C10—C8	179.2 (2)
C2—C1—C6	120.40 (18)	N4—C11—C12	120.61 (17)
C2—C1—Cl1	119.47 (15)	N4—C11—C16	123.50 (17)
C6—C1—Cl1	120.13 (17)	C12—C11—C16	115.88 (18)
C3—C2—C1	119.08 (19)	C13—C12—C11	121.7 (2)
C3—C2—H2A	120.5	C13—C12—H12	119.1
C1—C2—H2A	120.5	C11—C12—H12	119.1
C2—C3—C4	121.6 (2)	C12—C13—C14	121.1 (2)
C2—C3—Cl2	119.61 (17)	C12—C13—H13	119.5
C4—C3—Cl2	118.80 (17)	C14—C13—H13	119.5
C5—C4—C3	118.9 (2)	C15—C14—C13	119.1 (2)
C5—C4—H4A	120.5	C15—C14—H14	120.4
C3—C4—H4A	120.5	C13—C14—H14	120.4
C4—C5—C6	120.63 (19)	C14—C15—C16	120.4 (2)
C4—C5—H5	119.7	C14—C15—H15	119.8
C6—C5—H5	119.7	C16—C15—H15	119.8
C5—C6—C1	119.4 (2)	C15—C16—C11	121.82 (19)
C5—C6—N2	119.31 (17)	C15—C16—N5	117.01 (19)
C1—C6—N2	121.28 (17)	C11—C16—N5	121.12 (18)
			
C9—N1—N2—C7	0.7 (2)	N4—C7—C8—C9	−178.5 (2)
C9—N1—N2—C6	178.77 (16)	N2—C7—C8—C10	179.56 (19)
C6—C1—C2—C3	0.0 (3)	N4—C7—C8—C10	0.3 (4)
Cl1—C1—C2—C3	−179.77 (17)	N2—N1—C9—C8	−0.3 (2)
C1—C2—C3—C4	−0.1 (3)	C7—C8—C9—N1	−0.3 (2)
C1—C2—C3—Cl2	179.38 (17)	C10—C8—C9—N1	−179.1 (2)
C2—C3—C4—C5	0.3 (3)	C7—C8—C10—N3	45 (19)
Cl2—C3—C4—C5	−179.17 (17)	C9—C8—C10—N3	−136 (19)
C3—C4—C5—C6	−0.4 (3)	C7—N4—C11—C12	1.3 (3)
C4—C5—C6—C1	0.3 (3)	C7—N4—C11—C16	−177.99 (18)
C4—C5—C6—N2	178.21 (19)	N4—C11—C12—C13	−179.1 (2)
C2—C1—C6—C5	−0.1 (3)	C16—C11—C12—C13	0.2 (3)
Cl1—C1—C6—C5	179.68 (16)	C11—C12—C13—C14	0.5 (4)
C2—C1—C6—N2	−177.94 (18)	C12—C13—C14—C15	−0.8 (4)
Cl1—C1—C6—N2	1.8 (3)	C13—C14—C15—C16	0.3 (4)
C7—N2—C6—C5	110.9 (2)	C14—C15—C16—C11	0.4 (3)
N1—N2—C6—C5	−66.7 (2)	C14—C15—C16—N5	−177.1 (2)
C7—N2—C6—C1	−71.2 (3)	N4—C11—C16—C15	178.6 (2)
N1—N2—C6—C1	111.1 (2)	C12—C11—C16—C15	−0.7 (3)
N1—N2—C7—C8	−0.9 (2)	N4—C11—C16—N5	−3.9 (3)
C6—N2—C7—C8	−178.74 (17)	C12—C11—C16—N5	176.78 (18)
N1—N2—C7—N4	178.39 (17)	O1—N5—C16—C15	168.9 (2)
C6—N2—C7—N4	0.6 (3)	O2—N5—C16—C15	−10.8 (3)
C11—N4—C7—N2	−88.3 (2)	O1—N5—C16—C11	−8.7 (3)
C11—N4—C7—C8	90.8 (3)	O2—N5—C16—C11	171.6 (2)
N2—C7—C8—C9	0.7 (2)		

### Hydrogen-bond geometry (Å, º)

**Table:**

*D*—H···*A*	*D*—H	H···*A*	*D*···*A*	*D*—H···*A*
C9—H9···O2^i^	0.93	2.51	3.050 (3)	117
C5—H5···O1^ii^	0.93	2.58	3.374 (3)	143
N4—H4···N3^iii^	0.82 (3)	2.62 (3)	3.201 (3)	129 (2)
N4—H4···O1	0.82 (3)	2.02 (3)	2.608 (2)	128 (2)

Symmetry codes: (i) *x*, −*y*+1, *z*+1/2; (ii) −*x*+1/2, *y*−1/2, *z*; (iii) −*x*+1, −*y*+1, −*z*+1.
